# Nitrocellulose for Prolonged Permeation of Levofloxacin HCl-Salicylic Acid In Situ Gel

**DOI:** 10.3390/polym16070989

**Published:** 2024-04-04

**Authors:** Ei Mon Khaing, Kritamorn Jitrangsri, Parichart Chomto, Thawatchai Phaechamud

**Affiliations:** 1 Department of Industrial Pharmacy, Faculty of Pharmacy, Silpakorn University, Nakhon Pathom 73000, Thailand; khaing_e@silpakorn.edu (E.M.K.); chomto_p@su.ac.th (P.C.); 2 Department of Chemical Engineering and Pharmaceutical Chemistry, School of Engineering and Technology, Walailak University, Nakhon Srithammarat 80160, Thailand; kritamorn.ji@mail.wu.ac.th; 3 Natural Bioactive and Material for Health Promotion and Drug Delivery System Group (NBM), Faculty of Pharmacy, Silpakorn University, Nakhon Pathom 73000, Thailand

**Keywords:** nitrocellulose, in situ gel, levofloxacin HCl, salicylic acid

## Abstract

Currently, the application of solvent exchange-induced in situ gel is underway for drug delivery to the body target site. Nitrocellulose was attempted in this research as the matrix-forming agent in solvent exchange-induced in situ gel for acne and periodontitis treatments. The gel incorporated a combination of 1% *w*/*w* levofloxacin HCl and 2% *w*/*w* salicylic acid as the active compounds. In order to facilitate formulation development, the study explored the matrix-forming behavior of different concentrations of nitrocellulose in *N*-methyl pyrrolidone (NMP). Consequently, their physicochemical properties and matrix-forming behavior, as well as antimicrobial and anti-inflammatory activities, were evaluated using the agar cup diffusion method and thermal inhibition of protein denaturation in the egg albumin technique, respectively. All prepared formulations presented as clear solutions with Newtonian flow. Their contact angles on agarose gel were higher than on a glass slide due to matrix formation upon exposure to the aqueous phase of agarose, with an angle of less than 60° indicating good spreadability. Nitrocellulose concentrations exceeding 20% initiated stable opaque matrix formation upon contact with phosphate buffer pH 6.8. The high hardness and remaining force of the transformed gel indicated their robustness after solvent exchange. Fluorescence tracking using sodium fluorescein and Nile red confirmed the retardation of NMP and water diffusion by the nitrocellulose matrix. From the Franz cell permeation study, these drugs could permeate through neonate porcine skin and tissue of porcine buccal from the nitrocellulose in situ forming gel. Their accumulation in these tissues might enable the inhibition of the invading bacterial pathogens. The developed in situ gels effectively inhibited *Staphylococcus aureus*, *Staphylococcus epidermidis*, *Propionibacterium acnes*, and *Porphyromonas gingivalis*. Furthermore, the formulations demonstrated an anti-inflammatory effect. The low viscosity of LvSa25Nc makes it appropriate for injectable treatments targeting periodontitis, while the higher viscosity of LvSa40Nc renders it appropriate for topical applications in acne treatment. Therefore, the nitrocellulose in situ gel loaded with combined levofloxacin HCl and salicylic acid emerges as a promising dosage form for treating acne and periodontitis.

## 1. Introduction

Acne vulgaris (commonly called acne) is inflammatory and associates with the immune response to *Propionibacterium acnes*, a Gram-positive bacterium that colonizes sebum-rich follicles [1]. Among the various options, the administration of topical antimicrobial agents is the most widely favored therapeutic approach [2]. Conventional topical dosage forms and delivery systems are designed to achieve several objectives, including the normalization of shedding into the pore to prevent blockage, the elimination of *Propionibacterium acnes* and related microbes, induction of anti-inflammatory effects, and hormonal manipulation [3]. Novel carrier systems currently under investigation for the application and treatment of acne encompass liposomes, niosomes, microsponges, microemulsions, microspheres, solid lipid nanoparticles (SLNs), hydrogels, aerosols, fullerenes, and more [2,3]. Presently, only a limited number of drugs utilizing microsized or nanosized application systems have received approval for topical use and have been introduced to the market. Significant advancements have been achieved in recent years to enhance the performance of anti-acne care products.

Periodontitis is an inflammatory gum disease marked by the deterioration of periodontal ligaments, the development of periodontal pockets, and the resorption of the alveolar bone, leading to the breakdown of the supporting structure for the teeth. The emergence of various microflora, especially anaerobes such as *Porphyromonas gingivalis*, within these pockets is the primary cause of periodontitis [4]. Consequently, a local drug delivery system proves to be a valuable tool for local adjunctive pharmacological periodontal therapy. In contemporary times, a notable innovation in gel formulations is exemplified by in situ gels, which undergo a transition from a liquid to a semisolid state through a solvent exchange mechanism [5].

Currently, a solvent exchange-induced in situ gel is being employed for the delivery of drugs to periodontal pockets as a treatment for periodontitis. Atridox^®^ is a commercial product that contains doxycycline hyclate as an antibacterial agent, and this form of medication is administered to the patient through injection by a dentist [6]. Initially, the in situ gel comprises a solution containing polymers, organic solvents, and drugs. Upon injection into an aqueous environment, this solution solidifies into a polymeric matrix loaded with the drug. The formation of this matrix through a phase inversion process facilitates sustained and localized drug release over an extended period [7]. The in situ gel should demonstrate suitable viscosity, favorable gel formation, ease of injectability, and extended drug release while also effectively combating microbial activity against pertinent pathogens. The fluid characteristics of the solvent exchange-induced in situ gel in the solution state rely on the type of polymer and solvent used, thus emphasizing the importance of considering polymer type, its concentration, and its solvent in this pharmaceutical dosage form [5,7].

**Figure 1:**
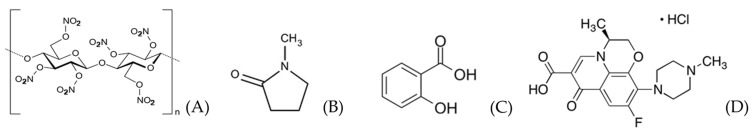
Chemical structures of nitrocellulose (**A**), N-methyl pyrrolidone (NMP) (**B**), salicylic acid (**C**), and levofloxacin HCl (**D**).

Nitrocellulose, a nitrated ester of cellulose, is a white, fibrous, pulp-like substance with no discernible odor or taste, characterized by the chemical formula C_12_H_16_N_4_O_18_ (Figure 1A). Nitrocellulose is employed in various applications, serving as a protective covering to prevent wound contamination in cosmetic preparations and for film production [8,9,10]. With its remarkable biological and physiochemical properties, nitrocellulose has been used in the form of a paper-like matrix featuring microscale porous pores, commonly referred to as a nitrocellulose membrane, which is frequently utilized to immobilize nucleic acids in Western blots [8,11]. Additionally, nitrocellulose bandages have proven effective in wound healing, especially for challenging-to-cover wounds [8]. Nano-porous nitrocellulose liquid bandages have been developed, exhibiting enhanced antibacterial effects, accelerated healing time, and non-toxicity to the wounded area itself [12]. Nitrocellulose, an aqueous insoluble nitrated ester of cellulose, exhibits solubility in esters, ketones, acetone, and glycol-ethers [9,10], making it unsuitable for pharmaceutical preparations. Our research revealed its solubility in N-methyl pyrrolidone (NMP) (Figure 1B), a typical solvent utilized in in situ gel dosage forms, including commercially available forms like Atridox^®^. Previously, moxifloxacin HCl-incorporated aqueous-induced Nc-based in situ gel was developed for periodontitis treatment using various solvents [13]. Only nitrocellulose at a concentration of 15% *w*/*w* was employed to modulate moxifloxacin HCl release. Different solvent types, including NMP, dimethyl sulfoxide, 2-pyrrolidone, and glycerol formal, influenced the sponge-like 3D structure of the dried nitrocellulose in situ gel remnants and affected mass loss during drug release [13]. However, there has been no prior report on the development and thorough investigation of in situ gel fabricated from nitrocellulose loaded with combined drugs for the treatment of acne and periodontitis.

Salicylic acid (Figure 1C) is present in various over-the-counter acne treatment products. Its efficacy in reducing acne lies in its ability to exfoliate the skin and maintain clear pores when applied topically. When salicylic acid is applied, it penetrates the skin, dissolving dead skin cells that may obstruct pores. Additionally, this compound exhibits anti-inflammatory, bacteriostatic, and antifungal properties by hindering the in vivo production of pro-inflammatory prostaglandins. This confers anti-inflammatory attributes, and it possesses both bacteriostatic and fungicidal properties [14]. Salicylic acid also functions to diminish redness and swelling (inflammation), thereby decreasing the formation of pimples and expediting the healing process of acne. Stearic acid–oleic acid nanoparticles in cream were designed for the controlled release of salicylic acid for topical application. The salicylic prolonged release could be achieved, thereby mitigating skin irritation, reducing the frequency of application, and ultimately enhancing patient compliance [15]. The gradual release of salicylic acid from Nc-based solvent exchange-induced in situ gel appears to align with the mentioned criteria for a suitable formulation in acne or periodontitis treatments.

*P. acnes* exhibits high resistance to azithromycin, SXT, erythromycin, and clindamycin while displaying significant susceptibility to minocycline, levofloxacin, and tetracycline [16,17]. A once-daily dose of 500 mg levofloxacin was well tolerated and may represent a promising antimicrobial option for acne therapy, given its elevated tissue levels and clinical effectiveness [18]. Levofloxacin (Figure 1D), a fluoroquinolone antibiotic with a broad spectrum, demonstrates activity against both Gram-positive and Gram-negative bacteria. The levofloxacin group showed a noteworthy decrease in acne score compared to the minocycline group, accompanied by a reduction in inflammatory lesion count [19,20]. In addition, this drug has also been currently of interest for use in periodontitis treatment due to its broad spectrum and good activity against selective anaerobic bacteria. Unique animal bite wound isolates such as *Porphyromonas macaccae*, *P. gingivalis*, and *Prevotella heparinolytica* are usually susceptible to 0.25 and 0.5 mg/mL of levofloxacin, respectively [21,22]. The combination of salicylic acid with levofloxacin is anticipated to improve the effectiveness of treatments for both acne and periodontitis, capitalizing on their unique mechanisms of action in antimicrobial activities. A dual-drug-loaded solvent-induced phase inversion-induced in situ-forming implant was fabricated to deliver pitavastatin (osteogenic drug) and tedizolid (antibiotic) using zein as the implant matrix for bone healing [23]. Additionally, the anti-inflammatory properties of salicylic acid are likely to alleviate redness and swelling associated with acne pimples and inflamed periodontal pockets. In order to inject into the periodontal pocket, an in situ gel for periodontal treatment should possess low viscosity, ensuring easy injectability and rapid phase inversion. On the other hand, for topical application on acne pimples, a more viscous gel is necessary. Therefore, the combined levofloxacin HCl and salicylic acid-loaded in situ gels comprising different nitrocellulose concentrations should be formulated to cater to the specific requirements of each proposed treatment.

This study aimed to develop the solvent exchange-induced in situ gel formulation using nitrocellulose as the matrix-forming agent for acne treatment and periodontitis treatment by loading combined levofloxacin HCl and salicylic acid as the active compounds. To achieve formulation development, understanding the matrix-forming behavior of nitrocellulose at different concentrations was investigated. Thus, the influence of nitrocellulose concentrations was studied and discussed in terms of physicochemical properties, matrix-forming behavior, and antimicrobial and anti-inflammatory activities.

## 2. Materials and Methods

### 2.1. Materials

Nitrocellulose (type RS ¼, lot no. 200906/09018) (viscosity of 3.0–5.9 s at 25.0% in acetone, nitrogen content of 11.77% with degree of substitution in NC of 3 and acid content maximum of 0.03%) graciously provided by Nitro Chemical Industry LTD., located in V.S. Chem House Bldg., Pathumwan, Bangkok, Thailand, was employed as the gel-forming agent. Levofloxacin HCl was kindly supplied by Siam Pharmaceutical Co., Bangkok, Thailand, and was used as an antimicrobial drug. Salicylic acid (Lot No. 0000525689) was procured from HiMedia Laboratories Pvt. Ltd., Thane, Maharashtra, India. NMP (Lot No. 144560-118, QReC, Auckland, New Zealand) served as the vehicle. Potassium dihydrogen orthophosphate (Lot No. E23W60) and sodium hydroxide (Lot No. AF310204) from Ajax FineChem, New South Wales, Australia, were dissolved in water to prepare PBS at pH 6.8. Acetronitrile (RCI Labscan, Bangkok, Thailand) (HPLC grade) was employed for HPLC analysis. Agarose (Lot No. H7014714, Vivantis, Selangor Darul Ehsan, Malaysia) was acquired to create an agarose gel. Sodium fluorescein (lot no. SHBL6563, Sigma, Livonia, MI, USA) and Nile red (lot no. BCBP8959V, Sigma, USA) were used for the study of microscopic interfacial behavior. Strains of *Staphylococcus aureus* ATCC 6538, *S. epidermidis* ATCC 5868 *and P. acnes* ATCC 14,916 were sourced from the Ministry of Public Health, Mueang, Nonthaburi District, Thailand. *P. gingivalis* ATCC 33,277 (Microbiologics Inc., St Cloud, MN, USA) was procured from Thai Can Biotech Co., Ltd., Bangkok, Thailand and also used as test microbes. Tryptic soy agar and tryptic soy broth (Difco^™^, Detroit, MI, USA) were used as media for antimicrobial investigations of the first three microbe strains. Sheep blood agar (Ministry of Public Health, Nonthaburi, Thailand) was used as a medium for *P. gingivalis*.

### 2.2. Preparation of In Situ Gel Formulations

The combined levofloxacin HCl and salicylic acid-loaded in situ gels comprising different nitrocellulose concentrations dissolved in NMP were prepared through a straightforward mixing method using a magnetic stirrer. Various concentrations of nitrocellulose (10, 15, 20, 25, 30, 35, and 40% *w*/*w*) and a constant 1% *w*/*w* levofloxacin HCl and 2% *w*/*w* salicylic acid were dissolved in NMP solvent at 25 °C while being stirred in a glass container. Control formulations included 1% *w*/*w* levofloxacin HCl in NMP (LvN), 2% *w*/*w* salicylic acid in NMP (SaN), and 2% *w*/*w* salicylic acid in NMP with 25% nitrocellulose (Sa25Nc). They were prepared using the same method as described earlier. Additionally, control groups consisted of 25% nitrocellulose in NMP (25Nc) formulation and NMP alone. The detailed composition of all formulations is provided in Table 1.

### 2.3. Evaluations

#### 2.3.1. Physical Appearance and Measurements of Viscosity and Rheology

The physical appearance of the prepared formulations was examined, taking into account factors such as clarity, color, and the presence of precipitates. Viscosity measurements of the formulations were carried out using a viscometer cone-plate RM 100 CP2000 plus (Lamy Rheology Instruments, Champagne-au-Mont-d’Or, France) at a temperature of 25 °C. These measurements were conducted in triplicate. Rheology measurements were also performed with the same instrument, varying the shear rate up to 1500 s^−1^.

#### 2.3.2. Contact Angle Determination

The sessile drop technique is commonly utilized to directly measure the contact angle, providing insights into the preferential wetting properties of the target surface with respect to the liquid droplet. Contact angle assessments were conducted using a drop shape analyzer (FTA 1000, First Ten Angstroms, Newark, CA, USA) through the sessile drop technique. The tests were performed on both a glass slide and the agarose gel surface, maintaining a pump-out rate of 1.9 µL/s through a 14-gauge needle. The angle was recorded at a time point of 5 s, and these measurements were carried out in triplicate.

#### 2.3.3. Gel Formation Study

The transition from a solution to a gel or solid matrix-like state through aqueous induction phase transition was assessed by injecting a 1 mL aliquot of the prepared formulation through an 18-gauge stainless needle into 5 mL of PBS (pH 6.8) and photographing the morphological changes at various intervals (1, 5, 10, 20, and 30 min).

#### 2.3.4. Study of Mechanical Properties

The study evaluated the mechanical properties of an in situ gel formulation after undergoing solvent exchange within a 7 mm diameter hollow agarose gel containing PBS (pH 6.8) for 3 days [24]. The gel’s behavior was tested using a texture analyzer (TA.XT plus, Stable Micro Systems, Surrey, UK), with a 5 mm diameter stainless spherical probe gradually pressed into the center of the gel. The relationship between applied force and probe displacement over time was measured, and maximum penetration force and remaining force were recorded (*n* = 3).

#### 2.3.5. Study of Microscopic Interfacial Behavior

Fluorescence tracking was employed to examine microscopic interfacial changes during the movement of NMP from the formulation and aqueous phase from agarose. The investigation involved a 0.4 μg/mL sodium fluorescence-loaded agarose gel and 0.25 μg/mL Nile red-loaded formulations or color-free formulation and agarose. The fluorescent color change at the interface was observed using an inverted fluorescent microscope (Nikon Eclipse TE2000S, Nikon, Kawasaki, Japan) at 40× magnification. A blue (B2A) filter excited at 450–490 nm visualized the green color of sodium fluorescein, while a green (G2A) filter with excitation of 510–560 nm tracked the red color of Nile red. Images were captured at 0, 2, 4, 6, 8, and 10 min to monitor interface changes.

#### 2.3.6. In Vitro Permeation Study

The levofloxacin HCl and salicylic acid contents in the prepared SaN, LvN, LvSa25Nc, and LvSa40Nc samples were assessed through high-performance liquid chromatography (HPLC) (Agilent 1260 Infinity, San Diego, CA, USA) at 264 nm with a Hypersil^TM^ BDS C18 column (250 × 4.6 mm, 5 µm particle size, Thermo Scientific, Waltham, MA, USA) (*n* = 6), following a standard curve analysis. The mobile phase consisted of phosphate buffer pH 4.7, methanol, and acetonitrile (85:3:12 *v*/*v*/*v*) at a flow rate of 1 mL/min at a temperature of 27 °C using a UV detector at 295 nm.

The fresh bulge tissues of pig and newborn pig skin were obtained from a local slaughterhouse in Nakhon Pathom province, Thailand. To prepare the buccal mucosa membrane, the subcutaneous fatty layer and connective tissues were removed from the protrusion of the porcine cheek tissue, followed by washing with PBS (pH 7.4). The newborn pig skins were from pigs that died naturally shortly after birth and were provided by the local slaughterhouse; subcutaneous fat was carefully removed.

The prepared buccal mucosa membrane and skin samples were stored at −4 °C and placed at 4 °C one day before the experiments. Two hours prior to the experiments, the skin was pre-equilibrated in phosphate buffer solution (PBS pH 7.4) at 25 °C. A circular specimen of 3 × 3 cm^2^ membrane and skin was then placed in a Franz diffusion cell, with the stratum corneum side facing the donor compartment for the skin specimen. The external part of the buccal membrane was placed facing the donor compartment. The receiver compartment (13 mL) was filled with PBS pH 5.5 for skin permeation and PBS pH 6.8 for buccal membrane permeation, maintained at 37 °C ± 1 °C and stirred magnetically. At various time intervals during 24 h, 2 mL of the solution from the receiver compartment was removed and substituted with the same volume of fresh medium. The drug permeation across pig skin and buccal membrane into the receptor fluid, as well as the residual amounts of levofloxacin HCl and salicylic acid in the donor compartment, pig skin, buccal membrane, and receptor fluid, were quantified using the aforementioned HPLC techniques (*n* = 3). The cumulative amount of salicylic acid and levofloxacin HCl that permeated through the newborn pig skin and buccal membrane per unit area was calculated from the concentration of each substance in the receiving medium per contact surface area of used skin or membrane and plotted as a function of time. The flux was determined as the slope of the linear portion of the plot. In addition, the lag time was determined from the linear equation of the plot.

#### 2.3.7. In Vitro Anti-Inflammatory Study

The effectiveness of in situ gel formulations (SaN, LvSa25Nc, and LvSa40Nc) in reducing inflammation was assessed by measuring their ability to inhibit the heat-induced denaturation of egg albumin. This process involved preparing a 5 mL solution consisting of 2.8 mL of PBS (pH 6.4), 0.2 mL of egg albumin from fresh hen’s eggs, and 2 mL of a sample from the Day 2 Franz cell reservoir of drug permeation through the porcine buccal membrane. The testing included negative and positive controls, which were a 0.9% normal saline solution and 750 mcg/mL diclofenac sodium, respectively. The procedure involved a 15 min incubation of the solutions at 37 °C, followed by heating at 70 °C for 5 min in a water bath. After cooling to room temperature, the absorbance of the solutions was measured at 660 nm with a UV–vis spectrophotometer (Hitachi U-2000, COAX Group Corporation Ltd., Bangkok, Thailand). The results were used to calculate the percentage inhibition of protein denaturation using Equation (1):

(1) $$\%inhibition=\frac{absorbance\;of\;negative\;control-absorbance\;of\;sample}{absorbance\;of\;negative\;control}\times 100$$

#### 2.3.8. Antimicrobial Activity Test

The antimicrobial properties of the prepared formulations were evaluated through the agar diffusion assay (cylinder plate method) against *S. aureus* ATCC 6538, *S. epidermidis* ATCC 5868, *P. acnes* ATCC 14,916, and *P. gingivalis* ATCC 33,277. This method involved the diffusion of the test sample from a stainless-steel cylinder cup (6 mm in diameter and 10 mm in height) through agar media inoculated with the test microbes. The microbial inocula in TSB, adjusted to a turbidity close to the 0.5 McFarland standard, were spread on TSA for *S. aureus* ATCC 6538, *S. epidermidis* ATCC 5868, and *P. acnes* ATCC 14,916. For *P. gingivalis*, the bacterial inocula with turbidity close to the 0.5 McFarland standard were spread on sheep blood agar.

A 100 µL aliquot of each solution formulation was filled into the cylinder cap using a micropipette, which was then positioned on the surface of an inoculated medium plate. Subsequently, the plates were incubated in the incubator (Thermo Scientific Precision Compact Incubators, Thermo Scientific, Cincinnati, OH, USA) for *S. aureus* ATCC 6538 and *S. epidermidis* ATCC 5868 and in an anaerobic incubator (Forma Anaerobic System, Thermo Scientific, Cincinnati, OH, USA) for *P. acnes* ATCC 14,916 and *P. gingivalis*. LvN and SaN were employed as positive control groups. Following a 24 h incubation at 37 °C, the inhibition zone diameter against these microbes was measured using a standard ruler (*n* = 3).

### 2.4. Statistical Analysis

The data presented were shown as the mean ± standard deviation (S.D.). To determine the significance of the findings, a one-way analysis of variance (ANOVA) was conducted, followed by the LSD post hoc test using SPSS software for Windows (version 11.5). Statistical significance was defined by a *p*-value of less than 0.05, indicating a notable difference in the results.

## 3. Results and Discussion

### 3.1. Physical Appearance and Viscosity

All prepared formulations appeared as yellowish transparent solutions similar to single drug-loaded solutions. The incomplete dissolved nitrocellulose was found at a concentration higher than 40% *w*/*w*. This feature has been previously documented when a greater amount of gel-forming agent is incorporated into the in situ gel formulation [5,24]. Before undergoing a phase transformation due to water induction, these in situ gels exist in a solution state. The loading capacity of the gel-forming agent relies on its solubility in the solvents utilized [5,13].

**Figure 2:**
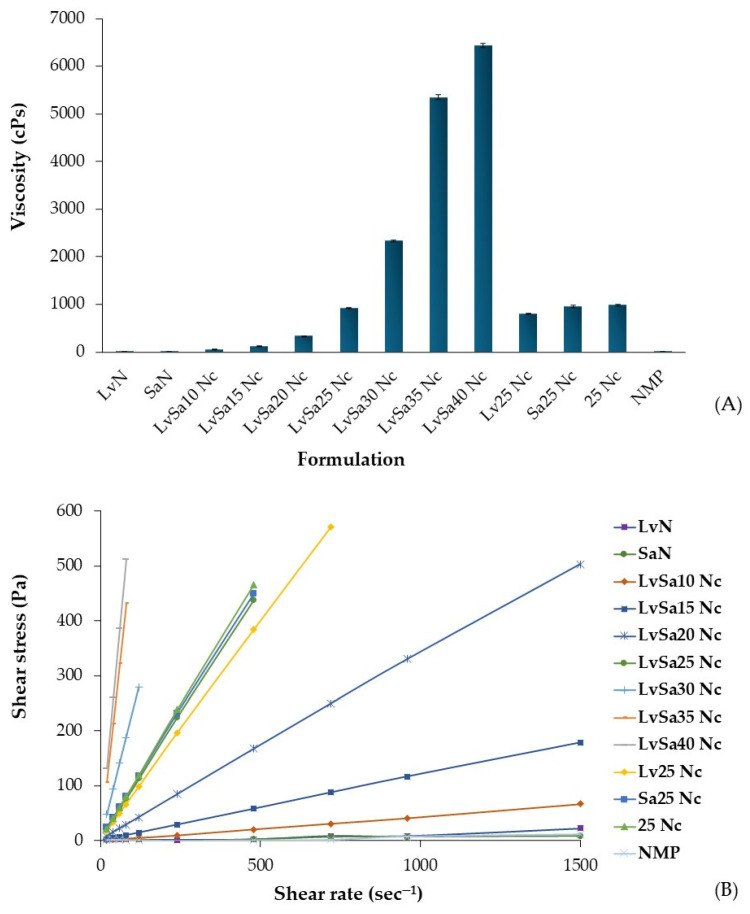
Viscosity (**A**) and plot of shear rate and shear stress (**B**) of levofloxacin HCl and salicylic acid-loaded nitrocellulose in situ gel formulations and control groups.

Additionally, formulations without drugs, with a single drug, or with combined drugs, all containing 25% *w*/*w* nitrocellulose, exhibited similar viscosity and rheology patterns, as illustrated in Figure 2A,B. Consequently, the incorporation of the drug did not significantly impact the viscosity of the formulation. The concentration of levofloxacin HCl at 1% *w*/*w* was used according to its efficient antimicrobial inhibition for periodontitis treatment, as previously reported [7]. Salicylic acid at 2% *w*/*w* has been employed for acne treatment [25,26]. All formulations demonstrated Newtonian flow, with the graph’s slope between shear rate and shear stress increasing at higher nitrocellulose concentrations (Figure 2B). This characteristic, exhibiting Newtonian flow and a steeper slope on the rheological graph, has been previously reported for injectable in situ gel dosage forms loaded with gamboge [24] and camphor [5]. In practical terms, an in situ gel formulation with lower viscosity is easier to apply via injection into the periodontal pocket [4]. Using smaller molecules as gel-forming agents, such as camphor, facilitated the enhancement of injectability in the in situ gel [5]. However, it is essential that the formulation contains enough gel-forming material or polymer to form a dense matrix, which is crucial for controlling drug release [13,24]. From this aspect of the investigation, it was notably observed that the viscosity of the prepared formulations significantly relied on the concentration of nitrocellulose used. Consequently, additional evaluations were carried out to examine other characteristics of the formulations after they transformed from solution into a gel or matrix-like mass.

### 3.2. Contact Angle

Besides the contact angle measurement on the glass slide surface for the spreadability check, this study examined the transformation of a solution into a gel state and a solid matrix-like mass on agarose surfaces, simulating the conditions of the gingival mucosa in periodontal pockets. The research utilized an agarose gel surface containing phosphate ions and water as a substitute substrate. Due to its smooth and flat characteristics, the agarose gel was suitable for assessing the contact angle of a nitrocellulose-based in situ gel formed by solvent exchange. This evaluation used drop shape analysis to visually determine contact angles. Table 2 displays the contact angles of all formulations on these surfaces. NMP thoroughly wetted the hydrophilic agarose surface from its high miscibility with the aqueous phase, resulting in a statistically significant lower contact angle compared to the glass slide (*p* < 0.05). Furthermore, the contact angle of NMP on these surfaces was significantly lower than that of nitrocellulose-loaded formulations (*p* < 0.05). Increasing nitrocellulose concentrations led to higher contact angles on the glass slide and agarose gel surfaces. Notably, contact angles on the agarose surface were statistically significantly higher than on the glass slide (*p* < 0.05), except for the LvSa40Nc and 25Nc formulations. As mentioned in the previous section, an increase in nitrocellulose content resulted in higher viscosity, consequently leading to reduced spreadability on these surfaces. Upon contact with the agarose surface, NMP diffused from the formulation, and the aqueous phase from the agarose gel triggered a phase inversion of the aqueous insoluble polymer, nitrocellulose, into a gel and solid polymeric matrix. This transition from a flowable liquid to a condensed droplet mass reduced spreadability and resulted in higher contact angles compared to that on the dry glass slide surface [13]. Previous research reported reduced spreadability of Eudragit L-based in situ gels using propylene glycol as a solvent from its phase transformation on agarose gel surface [27]. Despite higher contact angles for the developed in situ gels, they remained below 60°, indicating good wettability on test surfaces [27,28]. The significantly higher contact angle of LvSa40Nc on the glass slide compared to the agarose gel (*p* < 0.05) might be due to its high viscosity, which interfered with solvent exchange and phase inversion. Thus, the measurement of contact angle, especially on the surface of agarose gel, is an interesting technique to illustrate the spreadability of the phase-transformed in situ gel system.

### 3.3. Gel Formation

**Figure 3:**
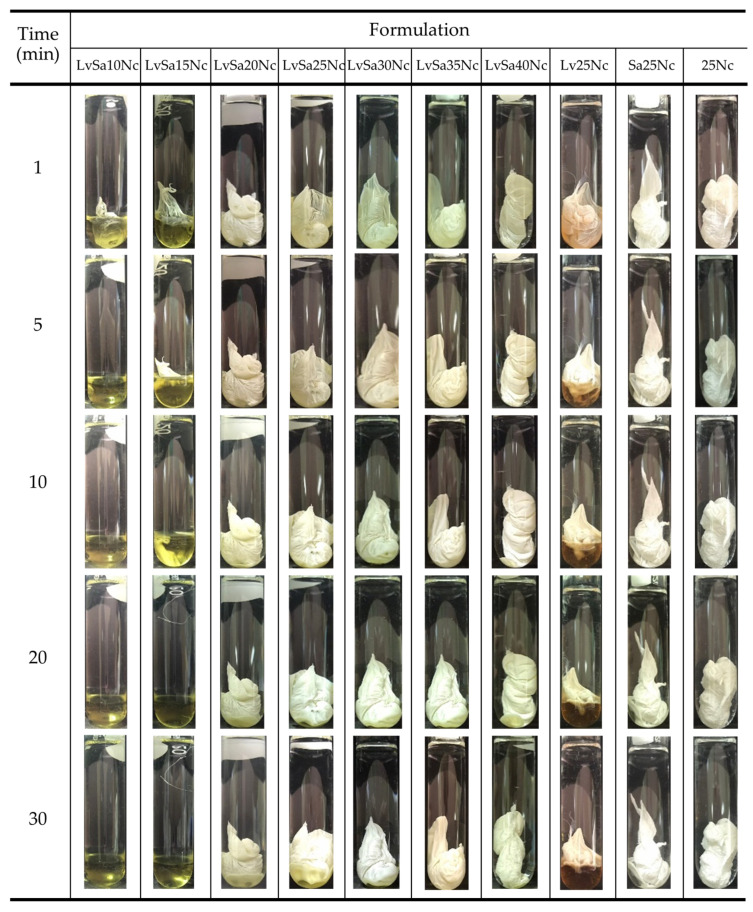
Change in matrix formation of levofloxacin HCl and salicylic acid-loaded nitrocellulose in situ gel formulations and control groups in PBS pH 6.8.

Aqueous phase-inducing phase transformation of prepared formulations was observed after they were injected into PBS pH 6.8, and the photos are shown in Figure 3. Formulations lacking nitrocellulose, including LvN and SaN, were compatible with this medium, exhibiting no phase separation. Formulations with a low concentration of nitrocellulose, specifically LvSa10Nc and LvSa15Nc, underwent a partial transition to an opaque gel mass, which eventually dissolved into the solution. The higher nitrocellulose content in LvSa15Nc extended the time required for the opaque matrix to fully dissolve compared to LvSa10Nc. Formulations with 20% *w*/*w* to 40% *w*/*w* nitrocellulose content experienced a rapid phase transformation upon exposure to the buffer, leading to the stabilization of the in situ gel. This process initiated the formation of a gel and, subsequently, a solid matrix, depending on the residual solvent [29,30]. The cloudy mass surrounding the formulation droplet, resembling skin, was initially formed, followed by a phase transformation that progressed towards the internal liquid formulation. Despite the expected delay in the formation of the surrounding cloudy mass due to the increased viscosity of formulations with higher nitrocellulose loading, as previously described in Figure 2A, the predominant cause of this phase separation was the denser polymer. Notably, at a 25% *w*/*w* nitrocellulose concentration, the lower layer Lv25Nc mass returned to liquid again. This phenomenon could be attributed to the initial rapid gel formation on the surrounding contact surface of the glass tube, which hindered aqueous penetration and was insufficient to sustain the phase inversion to an opaque mass, unlike with other formulations. Commercial products for treating periodontitis, such as Atridox^®^, consist of 36.7% poly(DL-lactide) (PLA) dissolved in 63.3% NMP [31]. Some research applied other polymers for modulating antibacterial compound release with polymer concentration less than 35% *w*/*w* for periodontitis treatment [13,27]. Adequate polymer content is crucial for managing drug release, but it should not result in a too-viscous formulation that cannot be injected through a needle. The fast transformation into a gel and matrix polymer enables minimizing the burst drug release. Thus, achieving an optimal balance between viscosity and polymer concentration is essential to develop an injectable in situ gel formulation for sustainable drug release. As the nitrocellulose content increased in the formulation, the formulation mass became more visibly convex and hardened, aligning with previously reported findings on contact angle and gel formation. Higher polymer concentrations help maintain the shape, akin to the behavior of shape-memory polymers [32]. A polymer chain exhibits various levels of intramolecular motion, with translational diffusion being the most basic, or zeroth-order, motion. This is because not only does diffusion occur, but other forms of motion also evolve as the concentration of the polymer increases. The change in internal motion transitions from intramolecular movements within a single polymer chain to cooperative diffusion, reflecting a collective movement [33]. The result of these gel formation experiments highlights the critical role of nitrocellulose concentration in influencing the morphological properties and phase inversion behavior of the in situ gel formulation.

### 3.4. Mechanical Properties

**Figure 4:**
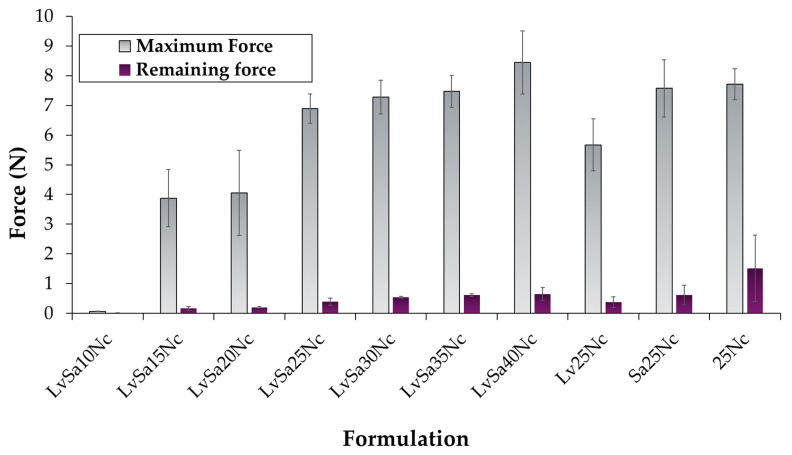
Mechanical property of levofloxacin HCl and salicylic acid-loaded nitrocellulose in situ gel formulations and control groups.

The mechanical properties of in situ gel are vital after undergoing phase inversion, to withstand jaw movements, and to preserve their shape within the periodontal pocket [34]. The maximum and remaining forces are also significant indicators of performance after the formulation has undergone transformation and been applied to treat acne. Figure 4 displays the maximum and remaining forces of the in situ gel formulation after it has fully transformed within a hollow agarose gel, following a complete solvent exchange into the matrix. Both the maximum penetration force and the remaining force exhibit an increasing trend as the concentration of nitrocellulose rises, indicating that the matrix becomes more robust due to the enhanced solidification of nitrocellulose. These observed parameters also aligned closely with their viscosity results, as depicted in Figure 2A. LvSa10Nc showed significantly lower values for both parameters, which could be attributed to its incomplete phase transformation. LvN and SaN were not evaluated for these properties since their formulations did not contain a polymer, similar to NMP. When comparing formulations with a 25% *w*/*w* nitrocellulose load, Lv25Nc demonstrated lower values for these parameters. Additionally, it appears that drug loading disrupts the polymeric matrix structure, leading to a decrease in the remaining force. The lowering maximum force of the matrix from transformed borneol-based in situ gel correlating with higher levels of drug incorporation has been reported previously [28]. Consequently, the mechanical properties of the transformed in situ gel showed a tendency to increase with dependence on the nitrocellulose concentration.

### 3.5. Fluorescent Tracking Matrix formation and Solvent Movement

**Figure 5:**
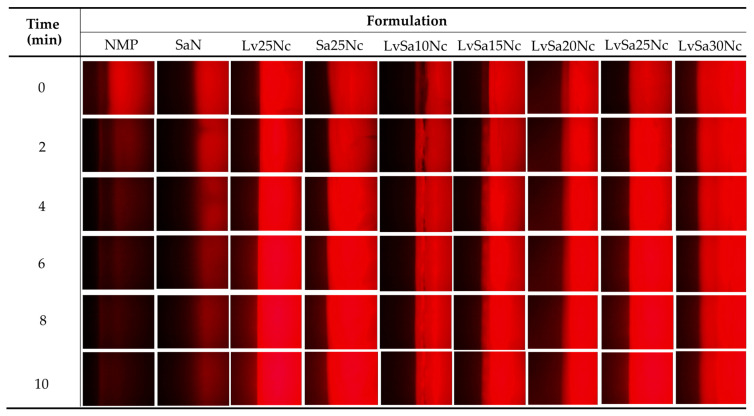
Interface interaction between noncolored agarose gel (**left**) against Nile red-loaded formulations (**right**) under an inverted fluorescent microscope at magnification of 40×.

**Figure 6:**
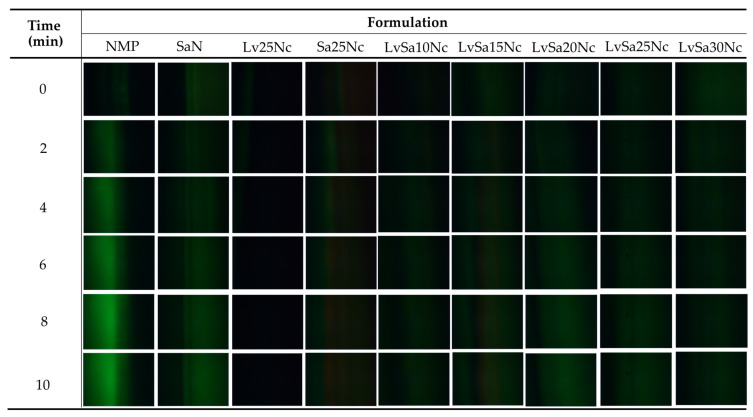
Interface interaction between noncolored agarose gel (**left**) against sodium fluorescence-loaded formulations (**right**) under an inverted fluorescent microscope at magnification of 40×.

The direct observation of solvent exchange of in situ gel systems is difficult. Some studies attempted HPLC for analysis of solvent movement of doxycycline hyclate-loaded bleached shellac-based in situ gel [35]. To better understand the formation of the nitrocellulose matrix and the movement of NMP/water, sodium fluorescein and Nile red tracking methods were adopted. Sodium fluorescein, absorbing blue light at 465–490 nm wavelengths, emits yellow-green fluorescence at 520–530 nm in water but does not emit in NMP [36]. Conversely, Nile red, a hydrophobic probe insoluble in water, shows intense red fluorescence in organic solvents and when dissolved in hydrophobic substances like lipids, but its fluorescence is quenched in water [37,38]. The observation following exposure between agarose gel (left) and Nile red-loaded in situ gel formulations (right) was undertaken under a fluorescent microscope and depicted in Figure 5. The Nile red dissolved in NMP and SaN apparently did not emit its red color after exposure in the agarose gel, indicating that the aqueous phase from the agarose gel diffused into NMP and SaN, and this fluorescent probe is quenched [39]. Initially, areas near the interface of a low nitrocellulose matrix formulation lost their intense red fluorescence, suggesting limited ability to prevent aqueous movement into the formulation. However, over time, the red fluorescence returned, indicating the formulation’s effectiveness in prohibiting further aqueous movement into the formulation. Formulations with a higher nitrocellulose content retained their red fluorescence, demonstrating the efficacy of the nitrocellulose matrix in preventing water phase diffusion. An interaction between the noncolored agarose gel and sodium fluorescein-loaded systems (Figure 6) showed the miscibility between NMP and the aqueous phase, allowing sodium fluorescein to diffuse from NMP (and partially from SaN) into the agarose gel, initiating green fluorescence emission on the left side while the nitrocellulose matrix prevented probe diffusion into the agarose gel.

**Figure 7:**
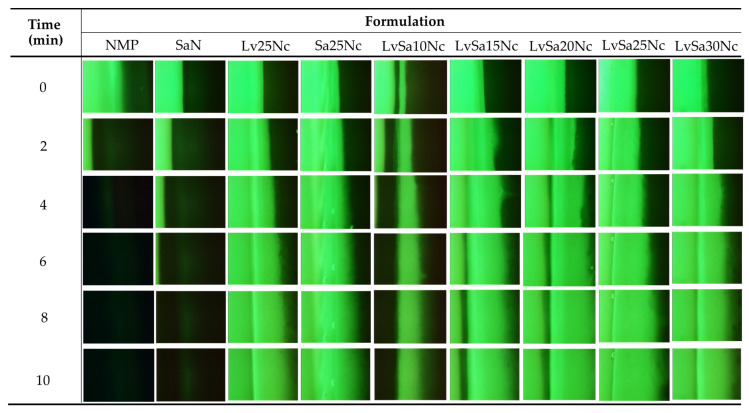
Interface interaction between sodium fluorescence-loaded agarose gel (**left**) against noncolored formulations (**right**) under an inverted fluorescent microscope at magnification of 40×.

The interaction at the interface between sodium fluorescein-loaded agarose gel and noncolored formulations is illustrated in Figure 7. This depiction shows the infiltration of emitted green fluorescence into nitrocellulose-based formulations, although certain areas, specifically in LvSa10Nc, experienced a fading of green fluorescence. Notably, NMP demonstrated a more rapid reduction in green fluorescence compared to SaN. These observations suggest a progressive transfer of the aqueous phase containing sodium fluorescein from the agarose gel into the formulations. The phenomenon where the larger volume of NMP, following swift mixing with the aqueous phase from the agarose gel, led to a decrease in green fluorescence emission underscores the dynamics of solvent exchange and the role of NMP in moderating fluorescence visibility [27].

**Figure 8:**
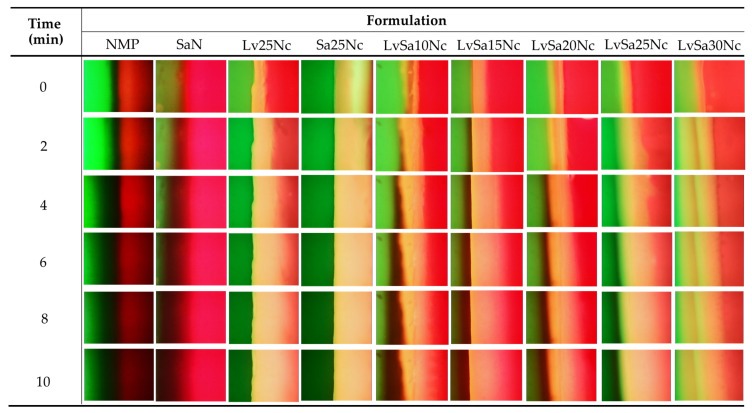
Interface interaction between sodium fluorescence-loaded agarose gel (**left**) against Nile red-loaded formulations (**right**) under an inverted fluorescent microscope at magnification of 40×.

The swift interaction and compatibility between NMP and the aqueous phase led to the absence of fluorescence emission for both sodium fluorescein and Nile red, as shown in Figure 8. This outcome is attributed to the increased hydrophilicity and polarity of the mixed solvent, potentially causing Nile red to precipitate and be quenched, while sodium fluorescein fails to sustain its green fluorescence when a significant quantity of NMP is combined with the aqueous phase [27]. In the case of SaN, an immediate decline in green fluorescence within the agarose gel was observed, although the red fluorescence remained detectable in the formulation. A notable decrease in both fluorescence indicators was particularly evident at the interface for in situ gels with 10–20% *w*/*w* nitrocellulose content. However, formulations containing up to 25% of this polymer displayed persistent fluorescence, indicating that an adequate concentration of nitrocellulose can create a matrix upon contact with agarose, thereby hindering NMP’s penetration into the agarose and diminishing the movement of the aqueous phase from the agarose into the formulation. The enhanced strength of the nitrocellulose matrix, as indicated in the mechanical properties as previously claimed (Figure 4), slowed down the solvent exchange process. The maximum force of LvSa25Nc and formulations containing 25% nitrocellulose abruptly increased when compared to that of LvSa20Nc. The employment of fluorescent probes like sodium fluorescein and Nile red is instrumental in examining and delineating the solvent exchange dynamics governed by the nitrocellulose matrix formation in in situ gel systems.

### 3.6. Drug Permeation

**Figure 9:**
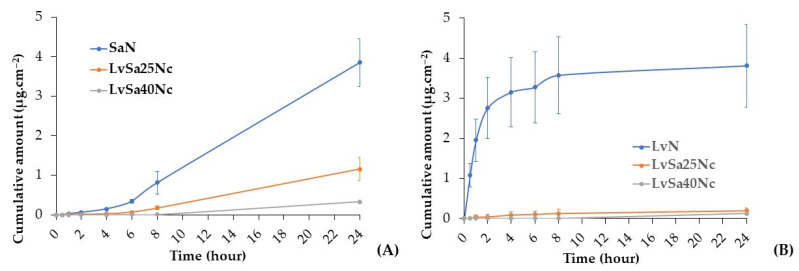
Permeation of salicylic acid (**A**) and levofloxacin HCl (**B**) through neonate porcine skin into PBS pH 5.5.

The content of levofloxacin HCl in the prepared LvN, LvSa25Nc, and LvSa40Nc was 102.12 ± 0.38%, 101.03 ± 0.81%, and 100.62 ± 0.49%, respectively. The content of salicylic acid of SaN, LvSa25Nc, and LvSa40Nc was 98.62 ± 0.38%, 100.52 ± 0.18%, and 99.62 ± 0.18%, respectively. This study conducted drug permeation testing using neonate porcine skin and buccal membrane separately. To simulate human skin pH, PBS with a pH of 5.5 was utilized as the receiver compartment, while PBS with a pH of 6.8 was employed as the receiver compartment for buccal membrane permeation. While PBS with a pH of 7.4 typically represents the pH of physiological or blood fluid, this study focused on the permeation primarily into the superficial tissue of both the skin and buccal membrane for these drug molecules. The salicylic acid levofloxacin HCl permeations through neonate porcine skin are reported in Figure 9. The permeation flux and lag times are detailed in Table 3, while Table 4 displays their accumulations in various sections of the Franz cell. The anaerobic bacterium *P. acnes* is considered crucial in the development of the pathophysiology of the common skin disease acne vulgaris. Some research detected high proportions of *P. acnes* DNA in enriched samples, particularly skin tissue-derived samples [40]. Cystic acne, a severe form of inflammatory acne, forms beneath the skin when pores are blocked by bacteria, dead skin cells, and oil [41]. This underscores the importance of studying drug penetration and accumulation in the skin from in situ gels. When comparing the penetration rates through neonate porcine skin, salicylic acid from SaN penetrated faster than LvSa25Nc and LvSa40Nc, as illustrated in Figure 9A. Furthermore, the flux value for salicylic acid was higher than those for LvSa25Nc and LvSa40Nc, as shown in Table 3. This indicates a slower permeation rate for the formulations with higher nitrocellulose loads, which is attributed to the denser nitrocellulose matrix, also resulting in longer lag times, as seen in Table 3. The prolonged drug permeation was confirmed by fluorescent probe tracking previously, which indicated greater retardation of solvent exchange due to an increased nitrocellulose matrix. The increase in retardation effect with higher polymer concentration has been previously reported, similar to testosterone permeation studies involving polyvinyl alcohol and polyvinylpyrrolidone films [42].

The freely permeated levofloxacin HCl from SaN through neonate porcine skin is evident, as shown in Figure 9B. NMP is effective in enhancing the drug permeation through the human epidermis by a cotransport mechanism [43], and this formulation had no nitrocellulose to retard drug permeation as found in LvSa25Nc and LvSa40Nc. At 24 h, salicylic acid exhibited greater permeation compared to levofloxacin HCl, largely due to the high lipophilicity of the salicylic acid molecule, which facilitates its permeation more effectively than levofloxacin HCl. The octanol–water partition coefficient (logP) of salicylic acid, measured using the traditional shake-flask method, was determined to be 2.35 [44], while the true partition coefficient of levofloxacin was 0.701 [45]. This difference aligns with the observation that salicylic acid accumulates more in skin tissue than levofloxacin HCl, as indicated in Table 4, across all formulations. Despite most of the drug remaining in the donor compartment, the presence of levofloxacin HCl in the tissue is beneficial for inhibiting bacteria like *P. acnes*, while salicylic acid significantly reduces inflammation. The remaining large amount of drug in the formulation could gradually diffuse and permeate through the skin tissue for prolonged drug transport. The prolonged permeation of salicylic acid is achieved, thereby mitigating skin irritation and diminishing the frequency of topical application, ultimately enhancing patient compliance [15]. The minute amount of these drugs in the receptor chamber was advantageous because the developed in situ gel was not intended for systemic drug delivery. Moisture retained on the skin after washing or bathing can trigger a phase transformation into a matrix, supporting sustained combined drug delivery for acne treatment. In addition, a viscous formulation such as LvSa40Nc should remain on the skin for acne treatment. The penetration of salicylic acid could enable the removal of dead skin cells that may obstruct pores. It has been reported to exhibit an anti-inflammatory effect by hindering the in vivo production of pro-inflammatory prostaglandins. Additionally, it is known to reduce inflammation by inhibiting the production of pro-inflammatory prostaglandins in vivo, offering anti-inflammatory benefits along with bacteriostatic and fungicidal properties [14].

**Figure 10:**
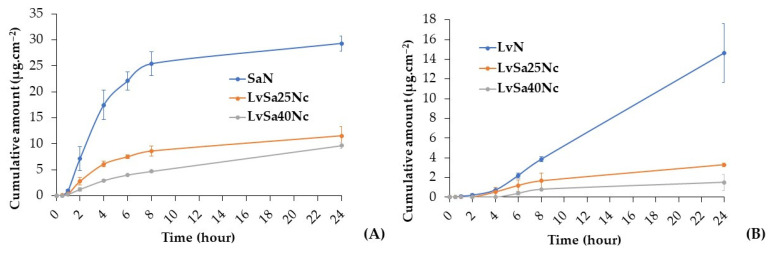
Permeation of salicylic acid (**A**) and levofloxacin HCl (**B**) through porcine buccal membrane into PBS pH 6.8.

Periodontitis is defined as a chronic inflammation of the periodontium, stemming from an imbalance between subgingival biofilms and the host’s susceptibility, leading to bacterial invasion and sustained infection in the gingival tissue, which, in turn, causes chronic inflammation [46,47]. For the purpose of studying drug permeation, this research utilized the porcine buccal membrane, chosen for its ease of preparation and the feasibility of stretching it between the donor and receptor chambers of the Franz diffusion cell. In a previous report, the modification of the Franz diffusion cell was required for using gingiva as barriers for transport study [48]. Additionally, assessing drug accumulation proved more feasible in the porcine buccal membrane compared to gingival tissue. According to Figure 10A, salicylic acid demonstrated significantly greater permeation through the porcine buccal membrane across all formulations, as evidenced by its higher Flux values in Table 3, compared to levofloxacin HCl. Salicylic acid also showed notably shorter lag times in permeation from all formulations compared to levofloxacin HCl, with a dependency on the concentration of nitrocellulose. This reinforces the earlier point that the high lipophilicity of the salicylic acid molecule enhances its permeation through the porcine buccal membrane more effectively than levofloxacin HCl.

The permeation profile of the nitrocellulose-free formulation demonstrated a steeper slope compared to LvSa25Nc and LvSa40Nc, resulting in a longer lag time, as indicated in Table 3, with the exception of levofloxacin permeation from LvSa40Nc through the porcine buccal membrane. The prolongation of drug penetration into the receptor compartment was still attained with nitrocellulose concentration dependence. As illustrated in Table 5, the accumulation of both drugs was higher in the donor compartment. Salicylic acid’s capacity for higher penetration through the buccal membrane into the buffer of the receptor compartment, relative to its accumulation in tissue, can be attributed to its high log *p*-value [44]. The accumulation of drugs in both the buccal tissue and the receptor was greater for SaN and LvN, respectively. This indicates that the denser nitrocellulose matrix with high mechanical properties, as reported previously, contributes to prolonging drug penetration. The drugs’ accumulation in the tissue highlights their potential to inhibit pathogens involved in periodontitis, particularly those affecting the superficial gingiva or pathogenic biofilms. In treating periodontitis, maintaining an effective drug concentration within the periodontal pockets is crucial for the therapy’s success [4,5]. Hence, the gradual diffusion of the remaining drug from the formulation into the crevicular fluid is essential for controlling and inhibiting pathogen growth in periodontitis treatment [47,48]. The effectiveness of the developed in situ gels against various microbes, including periodontitis pathogens, is further discussed in the subsequent section.

### 3.7. In Vitro Anti-Inflammatory Capabilities

**Figure 11:**
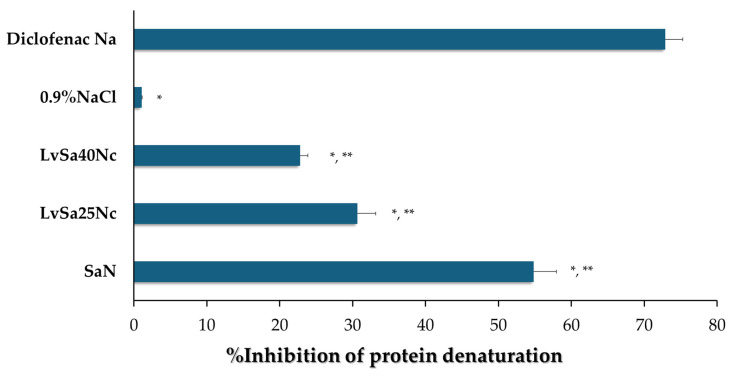
% Inhibition of protein denaturation of SaN, LvSa25Nc, LvSa40Nc, 0.9% NaCl, and 750 mcg/mL diclofenac sodium. The asterisk * symbol indicates a significant difference *(p* < 0.01) between three formulations and 0.9% NaCl, and ** symbol indicates a significant difference (*p* < 0.05) between three formulations by using one-way ANOVA followed by an LSD post hoc test.

The anti-inflammatory capabilities of SaN, LvSa25Nc, and LvSa40Nc were assessed through a method that measures the ability to prevent protein denaturation due to heat, specifically using egg albumin, following previously established protocols [49]. Results are illustrated in Figure 11. Thermal-induced protein denaturation occurs when heat or other external factors cause proteins to lose their tertiary and secondary structures. A lower rate of protein denaturation at elevated temperatures signifies stronger anti-inflammatory effects [50]. Among the tested formulations, LvSa40Nc showed a lower percentage of thermally induced protein denaturation compared to LvSa25Nc and SaN, indicating superior anti-inflammatory properties. The statistical analysis revealed significant differences in the percentage of protein denaturation inhibition between each pair (LvSa40Nc vs. LvSa25Nc and LvSa40Nc vs. SaN) with a *p*-value < 0.05. This suggests that modifying the diffusion of salicylic acid from a denser nitrocellulose matrix decreases its anti-inflammatory efficacy compared to the positive control, diclofenac sodium. The anti-inflammatory efficacy of these formulations correlated with the extent of salicylic acid permeation, as depicted in Figure 9 and Figure 10. Increasing the nitrocellulose loading in the formulation resulted in reduced salicylic acid permeation, consequently decreasing the anti-inflammatory effect. The anti-inflammatory benefits of NMP have been previously established [51], suggesting that combining it with salicylic acid could synergistically boost the anti-inflammatory effect, which is valuable for treating periodontitis, an inflammatory gum disease characterized by the destruction of periodontal ligaments [4]. Salicylic acid is renowned for its ability to inhibit prostaglandin synthesis, a critical anti-inflammatory mechanism, including the suggested inhibition of nuclear factor kappa B [52]. Protein denaturation can lead to the production of autoantigens associated with inflammatory conditions like rheumatic arthritis, cancer, and diabetes, implying that preventing protein denaturation can also reduce inflammatory responses [53]. Furthermore, the prolonged release of salicylic acid could reduce redness and swelling, aiding in the treatment of acne by preventing pimple formation and speeding up the healing process [15], enhancing skin tolerance, reducing application frequency, and improving patient compliance. Levofloxacin treatment impacts the production of TNF-α, a pro-inflammatory cytokine, and IL-10, an anti-inflammatory cytokine [54]. This effect could offer extra advantages in treating respiratory tract infections in pneumonic patients that are independent of its antibacterial properties. Thus, the findings of our investigation and the supplementary effect of the compounds used in our in situ gel underscore the potential benefits of these activities in alleviating acne and treating periodontitis.

### 3.8. Antibacterial Activities

**Figure 12:**
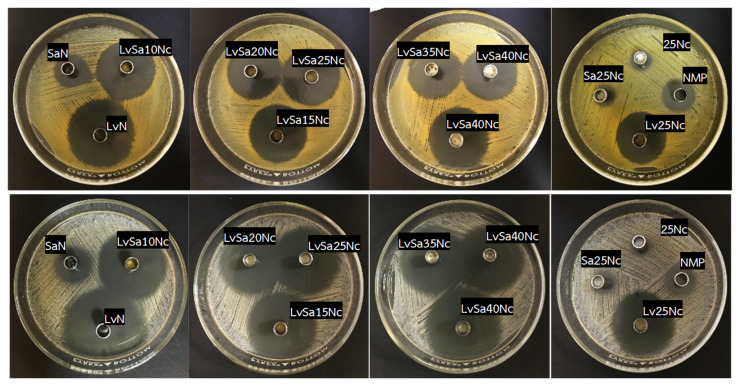
Photographs of the inhibition zone of levofloxacin HCl and salicylic acid-loaded nitrocellulose in situ gel formulations and control groups against *S. aureus* 6538 (**first row**) and *S. epidermidis* 5868 (**second row**).

**Figure 13:**
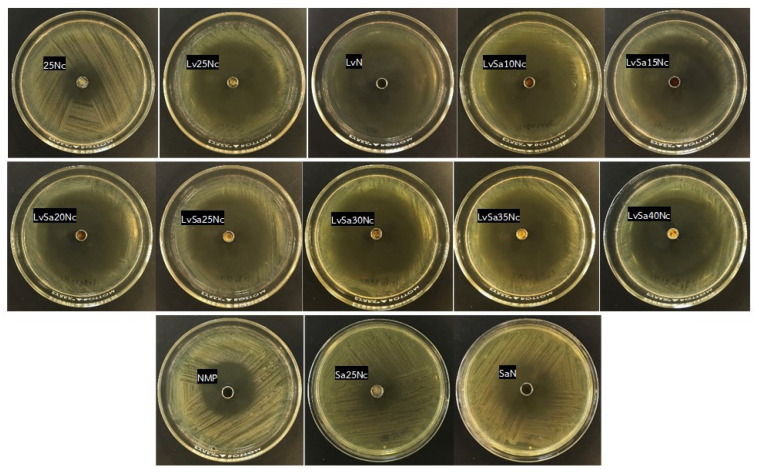
Photographs of the inhibition zone of levofloxacin HCl and salicylic acid-loaded nitrocellulose in situ gel formulations and control groups against *P. acnes* ATCC 14,916.

**Figure 14:**
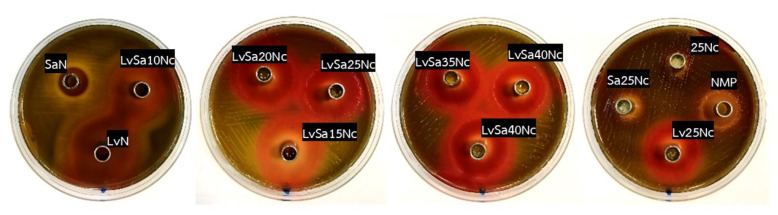
Photographs of the inhibition zone of levofloxacin HCl and salicylic acid-loaded nitrocellulose in situ gel formulations and control groups against *P. gingivalis* ATCC 33,277.

Table 6 and Figure 12, Figure 13 and Figure 14 display the antibacterial activities of the prepared formulations alongside control formulations against various bacteria, including *S. aureus* ATCC 6538, *E. epidermidis* ATCC 5868, *P. acnes* ATCC 14,916, and *P. gingivalis* ATCC 33,277. Comedones in acne are known to contain coagulase-negative staphylococci (*S. epidermidis*) and anaerobic diphtheroids (*P. acnes*) [2,3]. *S. aureus* and *P. gingivalis* are recognized pathogens associated with dental plaque or periodontal diseases. Specifically, *P. gingivalis* is highlighted in antimicrobial tests due to its role as a principal pathogen among anaerobic Gram-negative bacteria contributing to periodontitis [55,56]. Similarly, *S. aureus* has been identified in periodontal pockets of individuals with aggressive periodontitis [57]. In comparison tests, the LvN formulation exhibited has a statistically significant larger zone of inhibition against all tested microbes than the SaN formulation and others (*p* < 0.01), demonstrating superior efficacy. This enhanced activity is attributed to the salt form and high water solubility of levofloxacin HCl, which allows for its rapid release into the agar medium, effectively inhibiting bacterial growth more efficiently than salicylic acid, which relies on the broad-spectrum antibacterial properties of levofloxacin. Systemic levofloxacin as an adjunct to scaling and root planing has been previously mentioned as significantly improving both clinical and microbiological parameters in chronic periodontitis by a reduction in the percentage of sites positive for periodontopathic bacteria [58]. Salicylic acid, on the other hand, offers limited bacteriostatic and antifungal effects and works by penetrating the skin to dissolve dead skin cells that might block pores [14]. Salicylic acid has been shown to reduce lipogenesis in sebocytes by inhibiting the adenosine monophosphate-activated protein kinase (AMPK)/sterol response element-binding protein-1 (SREBP-1) pathway. Additionally, it diminishes inflammation by suppressing the NF-κB pathway in these cells [26]. The topical localized use of nitrocellulose-based in situ gel, both for acne and periodontitis applications, should be considered for safety, and further clinical safety determinations should be conducted. Nonetheless, the safety of nitrocellulose in cosmetic products such as nail lacquer has been addressed [59], and its extensive and lengthy polymeric chain [9,10] minimizes the likelihood of tissue penetration and resultant adverse effects.

The control formulation, which did not contain any drugs, displayed antibacterial effects against the tested microbes due to the antimicrobial properties of NMP. NMP-loaded poloxamer-based thermosensitive in situ gel exhibited antimicrobial activities against *S. aureus*, *E. coli*, and *C. albicans,* with the effectiveness of NMP being dependent on its concentration [60,61]. NMP is recognized as a suitable pharmaceutical solvent [62] and is used as a solubilizing agent in both parenteral and oral liquid formulations [63], which enables NMP to dissolve the lipids in cell membranes, leading to the disruption and leakage of microbial cell contents. Additionally, the presence of nitrocellulose in the formulation slowed the release of NMP into the agar medium. As a result, the inhibition zone produced by the 25Nc formulation was statistically significantly smaller than that produced by NMP alone (*p* < 0.01), yet it was comparable to the inhibition zone sizes observed for Sa25Nc.

As the concentration of nitrocellulose was increased from 10% *w*/*w* to 40% *w*/*w* in the formulation, a consistent decrease in the diameter of the inhibition zones against all tested microbes was observed (Table 6). This result corresponded with their lower contact angle, as presented previously in Table 2, owing to their higher viscosity and phase inversion. In addition, this reduction is attributed to the denser polymer matrix, which hinders the diffusion of the drug into the agar medium inoculated with bacteria. The drug’s release was gradual and extended due to increased tortuosity and reduced porosity, a result of the higher polymer content in the matrix [64]. This mechanism, which involves slowing down drug diffusion and resulting in smaller antimicrobial inhibition zones with higher polymer loading in the in situ gel, has been previously described [27,35]. Moreover, the salt form of levofloxacin, coupled with its high solubility in water, facilitated rapid diffusion out of the nitrocellulose matrix, leading to more effective inhibition of bacterial growth compared to salicylic acid.

The observation of a marginally larger inhibition zone for Sa25Nc compared to 25Nc suggests that salicylic acid possesses antibacterial properties. However, this effect was specifically noted against *P. gingivalis* ATCC 33,277 and *E. epidermidis* ATCC 5868. In contrast, the LvSa25Nc formulation exhibited a statistically significant larger inhibition zone than Lv25Nc (*p* < 0.05), indicating an enhanced antibacterial activity of the combined drugs against all tested bacteria (Table 6). This finding underscores the advantages of integrating levofloxacin HCl with salicylic acid in an in situ gel system, highlighting the synergistic benefits of this drug combination. To administer effectively into the periodontal pocket, a gel designed for periodontal care must have a low viscosity to allow for easy injection and swift transformation into a solid state. On the other hand, treating acne pimples topically requires a gel with higher viscosity. Therefore, the combined levofloxacin HCl and salicylic acid-loaded in situ gels comprising different nitrocellulose concentrations should be formulated to meet the distinct needs of each proposed treatment. Hence, the less viscous LvSa25Nc formulation is perfectly suited for injections used in treating periodontitis, while the viscous LvSa40Nc formulation is better suited for topical application on acne.

## 4. Conclusions

A solvent-exchange-induced in situ gel containing both levofloxacin HCl and salicylic acid was effectively formulated using nitrocellulose as the matrix-forming agent. All formulations exhibited Newtonian flow properties, with viscosity dependent on the loading of nitrocellulose. With increased nitrocellulose content, the gels showed enhanced matrix formation, resulting in a larger contact angle on agarose gel surfaces, yet maintained excellent spreading capabilities. Utilizing nitrocellulose concentrations above 20% *w*/*w* led to the stable formation of the matrix when exposed to aqueous environments. The gels’ increased hardness and residual force after phase change, dependent on the nitrocellulose concentration, underscored their enhanced robustness. Fluorescence probe emission studies with sodium fluorescein and Nile red demonstrated the nitrocellulose matrix’s capability of slowing down the diffusion of solvent and water. Levofloxacin HCl and salicylic acid were able to permeate through neonatal porcine skin and porcine buccal tissue from the gel, potentially allowing them to counteract invading bacterial pathogens in superficial tissue. These combined drug-loaded in situ gels successfully inhibited pathogens associated with periodontitis and acne with the gradual drug release from nitrocellulose matrix into inoculated agar. The evidence of drug accumulation in both test tissues confirmed the possibility of developing a dosage form to eliminate the invading pathogens. Additionally, their prevention of protein denaturation further indicates an anti-inflammatory effect. The low viscosity of LvSa25Nc renders it suitable for injectable treatments aimed at addressing periodontitis, whereas the higher viscosity of LvSa40Nc makes it suitable for topical applications in acne treatment. Consequently, this nitrocellulose-based in situ gel, loaded with levofloxacin HCl and salicylic acid, stands out as a promising treatment option for acne and periodontitis, offering sustained drug delivery and effective microbial inhibition.

## Figures and Tables

**Table 1:** Compositions of levofloxacin HCl and salicylic acid-loaded nitrocellulose in situ gel formulations.

Formulation	Concentration (% *w*/*w*)			
	Levofloxacin HCl	Salicylic Acid	Nitrocellulose	NMP
LvN	1			99
SaN		2		98
LvSa10Nc	1	2	10	87
LvSa15Nc	1	2	15	82
LvSa20Nc	1	2	20	77
LvSa25Nc	1	2	25	72
LvSa30Nc	1	2	30	67
LvSa35Nc	1	2	35	62
LvSa40Nc	1	2	40	57
Lv25Nc	1	2	25	74
Sa25Nc		2	25	73
25Nc			25	75
NMP				100

**Table 2:** Contact angle of levofloxacin HCl and salicylic acid-loaded nitrocellulose in situ gel formulations and control groups on glass slide and agarose gel surfaces.

Formula	Contact Angle ± S.D. (Degree) (*n* = 3)	
	Glass Slide	Agarose
LvSa10Nc	12.68 ± 2.32 ^b^	18.71 ± 2.82 ^b^
LvSa15Nc	12.88 ±1.12 ^c^	17.13 ± 2.70 ^c^
LvSa20Nc	15.15 ± 2.93 ^d^	22.04 ± 2.02 ^d^
LvSa25Nc	21.48 ± 2.98 ^e^	32.71 ± 3.32 ^e^
LvSa30Nc	30.72 ± 4.62 ^f^	42.80 ± 2.09 ^f^
LvSa35Nc	46.37 ± 2.33 ^g^	50.82 ± 0.16 ^g^
LvSa40Nc	58.44 ± 0.30 ^h^	51.41 ± 2.76 ^h^
Lv25Nc	25.07 ± 2.72 ^i^	28.98 ± 2.02 ^i^
Sa25Nc	35.23 ± 1.29 ^j^	46.17 ± 1.85 ^j^
25Nc	30.98 ± 1.00	33.81 ± 2.0
NMP	9.06 ± 3.69 ^a^	3.45 ± 0.23 ^a^

The superscripts a–j between columns represent significant difference (*p* < 0.05).

**Table 3:** Flux and lag time of salicylic acid and levofloxacin HCl permeation through neonate porcine skin and porcine buccal membrane.

Formulation	Neonate Porcine Skin		Porcine Buccal Membrane	
	Flux (µg/cm^2^/min)	Lag Time (min)	Flux (µg/cm^2^/min)	Lag Time (min)
Salicylic acid				
SaN	0.0030 ± 0.0017	235.29 ± 42.88	0.0907 ± 0.0128	46.35 ± 6.42
LvSa25Nc	0.0010 ± 0.0003	297.78 ± 15.97	0.0322 ± 0.0018	39.40 ± 12.47
LvSa40Nc	0.0003 ± 0.0001	335.67 ± 62.10	0.0127 ± 0.0001	26.81 ± 11.60
Levofloxacin HCl				
SaN	0.0178 ± 0.0150	95.31 ± 102.41	0.0415 ± 0.0546	162.01 ± 26.49
LvSa25Nc	0.0003 ± 0.0001	321.34 ± 170.65	0.0047 ± 0.0017	131.66 ± 93.87
LvSa40Nc	0.0001 ± 0.0000	606.00 ± 57.16	0.0009 ± 0.0008	1856.00 ± 193.28

**Table 4:** Salicylic acid (A) and levofloxacin HCl (B) remaining from donor chamber, neonate porcine skin, and receptor chamber of SaN, LvSa25Nc, and LcSa40Nc formulations after release test.

**(A)**						
**Compartment**	**SaN**		**LvSa25Nc**		**LvSa40Nc**	
	**(µg)**	**(%)**	**(µg)**	**(%)**	**(µg)**	**(%)**
Donor chamber	4499.07 ± 1316.10	80.38	4518.37 ± 442.07	67.98	6287.37 ± 584.51	93.93
Skin membrane	898.01 ± 53.84	16.04	2052.83 ± 224.75	30.88	384.97 ± 91.38	5.75
Receptor chamber	199.83 ± 39.92	3.57	75.65 ± 12.21	1.14	21.53 ± 1.74	0.32
**(B)**						
**Compartment**	**SaN**		**LvSa25Nc**		**LvSa40Nc**	
	**(µg)**	**(%)**	**(µg)**	**(%)**	**(µg)**	**(%)**
Donor chamber	2600.58 ± 167.32	84.91	2811.30 ± 234.81	84.60	3272.21 ± 271.96	97.82
Skin membrane	345.38 ± 31.68	11.31	505.75 ± 91.61	15.20	69.08 ± 0.8983	2.07
Receptor chamber	115.82 ± 94.10	3.78	6.71 ± 2.58	0.20	3.98 ± 0.1145	0.12

**Table 5:** Salicylic acid (A) and levofloxacin HCl (B) remaining in the donor chamber, buccal membrane, and receptor chamber of SaN, LvSa25Nc, and LcSa40Nc formulations after release test.

**(A)**						
**Compartment**	**SaN**		**LvSa25Nc**		**LvSa40Nc**	
	**(µg)**	**(%)**	**(µg)**	**(%)**	**(µg)**	**(%)**
Donor chamber	4171.27 ± 278.80	63.15	5229.84 ± 323.23	81.82	5170.67 ± 56.90	84.73
Buccal membrane	500.37 ± 57.34	7.58	424.71 ± 275.63	6.64	341.97 ± 153.71	5.60
Receptor chamber	1933.42 ± 18.22	29.27	736.98 ± 130.23	11.53	590.19 ± 38.92	9.67
**(B)**						
**Compartment**	**SaN**		**LvSa25Nc**		**LvSa40Nc**	
	**(µg)**	**(%)**	**(µg)**	**(%)**	**(µg)**	**(%)**
Donor chamber	1814.35 ± 190.44	60.67	2889.29 ± 154.51	90.40	2958.68 ± 82.50	97.01
Buccal membrane	735.80 ± 93.30	24.60	201.64 ± 124.58	6.31	46.11 ± 8.83	1.51
Receptor chamber	440.59 ± 111.67	14.73	105.15 ± 3.30	3.29	45.10 ± 23.50	1.48

**Table 6:** Zone of inhibition of levofloxacin HCl and salicylic acid-loaded nitrocellulose in situ gel formulations and control groups against different microbes (*n* = 3).

Formula	Inhibition Zone ± S.D. (mm)			
	*S. aureus* ATCC 6538	*E. epidermidis* ATCC 5868	*P. acnes* ATCC 14,916	*P. gingivalis* ATCC 33,277
LvN	37.3 ± 1.2	45.0 ± 1.7	73.1 ± 1.1	34.9 ± 0.8
SaN	18.3 ± 1.5	18.3 ± 0.6	29.7 ± 0.3	19.1 ± 0.2
LvSa10Nc	34.3 ± 0.6	40.0 ± 17	66.3 ± 2.0	33.7 ± 1.5
LvSa15Nc	33.0 ± 0.0	38.0 ± 1.0	64.4 ± 0.9	31.7 ± 1.9
LvSa20Nc	32.7 ± 0.6	38.3 ± 0.6	63.7 ± 0.6	29.0 ± 1.8
LvSa25Nc	33.3 ± 0.6 ^e^	39.0 ± 1.0 ^f^	62.3 ± 2.3 ^g^	27.8 ± 0.7 ^h^
LvSa30Nc	32.3 ± 0.6	39.7 ± 0.6	62.3 ± 0.5	27.6 ± 0.4
LvSa35Nc	31.7 ± 0.6	38.3 ± 0.6	61.7 ± 1.2	27.5 ± 0.5
LvSa40Nc	30.3 ± 0.6	38.3 ± 1.2	58.6 ± 1.3	26.4 ± 0.5
Lv25Nc	28.3 ± 0.6 ^e^	34.7 ± 0.6 ^f^	57.2 ± 0.2 ^g^	25.0 ± 0.2 ^h^
Sa25Nc	10.7 ± 0.6	13.3 ± 0.6	20.2 ± 0.5	11.9 ± 0.1
25Nc	10.8 ± 0.8 ^a^	10.3 ± 0.6 ^b^	26.3 ± 0.4 ^c^	10.3 ± 0.6 ^d^
NMP	16.3 ± 0.6 ^a^	16.8 ± 1.3 ^b^	38.2 ± 0.2 ^c^	18.0 ± 0.0 ^d^

The superscripts a–d and e–h in the same column represent a significant difference at *p* < 0.01 and *p* < 0.05, respectively, within the tested formulations. The superscripts e–h between columns represent significant differences (*p* < 0.05).

## Data Availability

Data are contained within the article.
